# Nanosize Non‐Viral Gene Therapy Reverses Senescence Reprograming Driven by PBRM1 Deficiency to Suppress iCCA Progression

**DOI:** 10.1002/advs.202414525

**Published:** 2025-01-17

**Authors:** Xiwen Wu, Yi Zhang, Yuan Ding, Jiali Yang, Zimin Song, Shuirong Lin, Ruhe Zhang, Jun Wu, Shunli Shen

**Affiliations:** ^1^ Department of Hepatic Surgery Center of Hepato‐Pancreato‐Biliary Surgery The First Affiliated Hospital of Sun Yat‐sen University Guangzhou Guangdong 510080 China; ^2^ Department of Clinical Nutrition Sun Yat‐sen University Cancer Center State Key Laboratory of Oncology in South China Guangdong Provincial Clinical Research Center for Cancer Collaborative Innovation Center for Cancer Medicine Guangzhou 510060 China; ^3^ Department of Hepatobiliary Surgery The Third Affiliated Hospital of Sun Yat‐sen University Guangzhou 510630 China; ^4^ Department of Hematology The Seventh Affiliated Hospital of Sun Yat‐sen University Shenzhen 518107 China; ^5^ Bioscience and Biomedical Engineering Thrust The Hong Kong University of Science and Technology (Guangzhou) Nansha Guangzhou Guangdong 511400 China; ^6^ Division of Life Science The Hong Kong University of Science and Technology Hong Kong SAR 999077 China

**Keywords:** ERK1/2, gene therapy, intrahepatic cholangiocarcinoma, nanomedicine, PBRM1, senescence reprograming

## Abstract

Polybromo‐1 (PBRM1) serves as a crucial regulator of gene transcription in various tumors, including intrahepatic cholangiocarcinoma (iCCA). However, the exact role of PBRM1 in iCCA and the mechanism by which it regulates downstream target genes remain unclear. This research has revealed that PBRM1 is significantly downregulated in iCCA tissues, and this reduced expression is linked to aggressive clinicopathological features and a poor prognosis. Furthermore, it is demonstrated that PBRM1 can impede iCCA progression, and a gene therapy nanomedicine is developed to treat iCCA in vivo by modulating PBRM1 expression. The heightened expression of PBRM1 induces by the nanomedicine substantially inhibited tumor growth in iCCA. Conversely, the decrease in PBRM1 results in the abnormal activation of the ERK1/2 signaling pathway, a reduction in p16, p53/p21, and cellular senescence, thereby promoting iCCA advancement. Treatment with U0126, an ERK1/2 inhibitor, effectively halted iCCA progression by regulating the PBRM1‐ERK1/2‐cellular senescence pathway. These findings underscore the significant role of PBRM1 in controlling iCCA progression and predicting prognosis. Targeting the PBRM1‐ERK1/2‐cellular senescence pathway with U0126 shows promise for clinical applications in treating iCCA.

## Graphical abstract

This research demonstrates that PBRM1 has the capability to inhibit the progression of intrahepatic cholangiocarcinoma (iCCA). A novel gene therapy nanomedicine is developed to treat iCCA in vivo by modulating PBRM1 expression, highlighting its crucial role in controlling iCCA and predicting prognosis. Furthermore, targeting the PBRM1‐ERK1/2‐cellular senescence pathway with U0126 presents a promising approach for clinical applications aimed at treating iCCA.

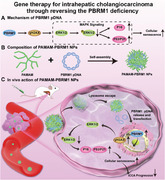

## 1 Introduction

Intrahepatic cholangiocarcinoma (iCCA) originating above the second‐degree bile duct is a prevalent primary liver cancer and the least common form of cholangiocarcinoma.^[^ ^1^, ^2^ ^]^ As one of the most devastating tumors, iCCA has been on the rise globally.^[^ ^1^, ^2^, ^3^ ^]^ Unlike hepatocellular carcinoma (HCC), most iCCA develops in non‐cirrhotic livers and no risk factors have been identified, making it hard to diagnose at an early stage.^[^ ^1^, ^2^ ^]^ By the time most iCCA patients are diagnosed, they are already in advanced stages, missing the opportunity for curative resection.^[^ ^1^, ^4^, ^5^ ^]^ Two FDA‐approved treatments (FGFR2 inhibitors and IDH inhibitors) have made certain progress in phase II and III clinical studies, respectively.^[^ ^6^, ^7^ ^]^ It is necessary to explore new therapeutic targets because this part of the cases is relatively small. The global increase in iCCA cases emphasizes the need to understand the molecular mechanisms underlying iCCA development and progression to pave the way for innovative treatments. Recently, non‐viral nanocarriers have recently garnered increasing attention for gene therapy in solid tumors due to their unique biosafety and efficient in vivo delivery advantages.^[^ ^8^, ^9^ ^]^ Nanomedicine composed of positively charged nanocarriers and loaded genes demonstrates promising treatment efficacy and holds high therapeutic potential for iCCA treatment.^[^ ^10^, ^11^, ^12^ ^]^ Among positively charged nanocarriers, polyamidoamine (PAMAM) has been the hotspot of recent gene delivery research due to its high gene recombination efficiency and low toxicity in vivo.^[^ ^13^, ^14^, ^15^ ^]^

SWI/SNF complexes modulate gene expression through chromatin remodeling.^[^ ^16^, ^17^ ^]^ The main difference between the SWI/SNF B (PBAF) complex and the SWI/SNF A complex is that it contains polybromo‐1 (PBRM1).^[^ ^16^, ^18^, ^19^ ^]^ The PBRM1 protein (also known as BAF180) contains 6 bromodomains (BDs) and 2 bromo‐adjacent homology (BAH) domains.^[^ ^19^ ^]^ Common mutations affecting PBRM1 include nonsense mutations, missense mutations, and loss of copy number, resulting in protein expression loss. As such, as a chromatin remodeling factor, PBRM1 plays a pivotal role in gene transcription regulation.

Recent exome sequencing studies have unveiled a high prevalence of SWI/SNF subunit mutations across various cancers, with frequent occurrences of PBRM1 mutations.^[^ ^16^, ^20^ ^]^ Mutations and loss of PBRM1 function have been identified in multiple tumors, with PBRM1 deletion or inactivation correlating with tumor progression, metastasis, and poor prognosis. ^[^ ^16^, ^21^, ^22^, ^23^ ^]^ PBRM1 also contributes significantly to cell cycle regulation by modulating p21 expression in response to diverse environmental stimuli.^[^ ^16^, ^24^ ^]^ PBRM1 deletion has been shown to decrease IFNγ‐STAT1 signaling in murine and human renal cell carcinoma (RCC) cell lines.^[^ ^25^ ^]^ Inactivation of PBRM1 was found to be associated with a lower immunogenic tumor microenvironment (TME) and immunotherapy resistance in an immunocompetent murine RCC model.

Recent exome sequencing has highlighted frequent PBRM1 mutations in iCCA cases.^[^ ^26^, ^27^ ^]^ Complete loss of breast cancer type 1 susceptibility protein (BRCA1)‐associated protein‐1 (BAP‐1) has been observed in 19.4% of iCCA cases, while loss of PBRM1 is present in 23.1% of cases.^[^ ^28^ ^]^ Exon sequencing of 32 iCCA cases revealed inactivating mutations in several chromatin remodeling genes, including PBRM1, ARID1a, and BAP1, with about half of the tumors showing at least one inactivating mutation in these genes.^[^ ^26^ ^]^ Moreover, retained PBRM1 expression is linked to poorer overall survival (OS) and disease‐free survival (DFS) in iCCA patients. Claudio Luchini et al. found that PBRM1 mutations are a late event during cholangiocarcinogenesis and these results have implications for the functional role of chromatin remodeling genes in biliary tumorigenesis.^[^ ^29^ ^]^ However, the precise role and function of PBRM1 in iCCA remain inadequately understood.^[^ ^30^ ^]^

**Scheme 1:**
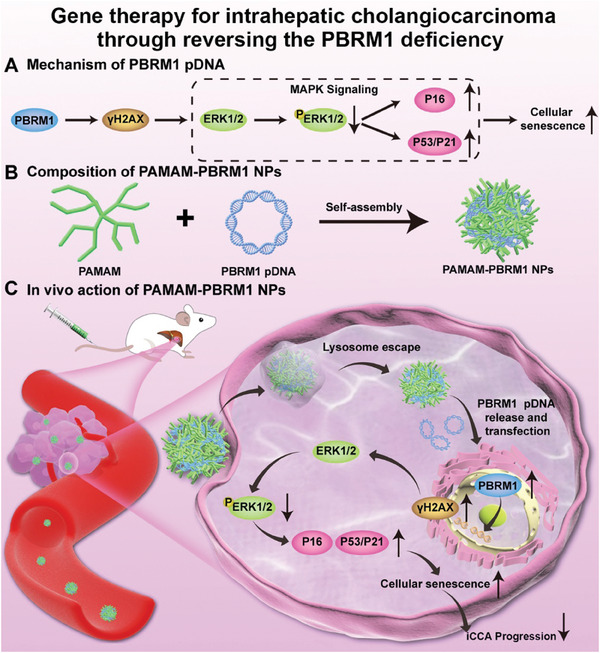
Schematic illustration of gene therapy for intrahepatic cholangiocarcinoma through reversing the PBRM1 deficiency. A) Mechanism of PBRM1 pDNA. B) Composition of PAMAM‐PBRM1 NPs. C) In vivo action of PAMAM‐PBRM1 NPs.

The present study aimed to investigate the correlation of PBRM1 with the clinicopathological characteristics and prognosis of iCCA patients. Additionally, a reliable gene therapy nanomedicine has been developed for in vivo cholangiocarcinoma treatment through the modulation of PBRM1 expression (**Scheme** **1**).

## 2 Results

### 2.1 PBRM1 Is Down‐Regulated in iCCA

**Figure 1:**
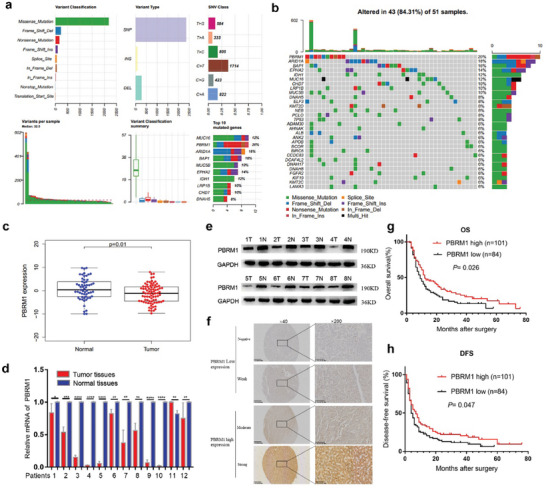
PBRM1 is downregulated in human iCCA and correlated with poor survival. a) Summary of mutation types of TCGA cholangiocarcinoma dataset. b) The waterfall plot shows the mutated genes in cholangiocarcinoma from the TCGA cohort. The right bar chart shows the Top 30 mutant genes frequencies of mutation. c) Expression level of PBRM1 in iCCA tissues and normal tissues (in GSE76297). d) The mRNA expression of PBRM1 in 12 paired iCCA tumors and normal tissues. e) The protein expression of PBRM1 in 8 paired iCCA tumor (T) and normal tissues (N). f) Representative immunohistochemical staining image of PBRM1 in iCCA. (g/h) Kaplan‐Meier curves for the overall survival and disease‐free survival of 185 iCCA patients according to the expression of PBRM1. Abbreviations: iCCA, intrahepatic cholangiocarcinoma. ^*^ *p* < 0.05, ^**^ *p* < 0.01, ^***^ *p* < 0.001, ^****^ *p* < 0.0001.

Using The Cancer Genome Atlas (TCGA) database, a waterfall plot analysis revealed that PBRM1 is the most frequently mutated gene in cholangiocarcinoma (**Figure** **1**a,b). Data from the Gene Expression Omnibus (GEO) database (GSE76297) demonstrated a significant reduction in PBRM1 messenger (mRNA) expression in tumor tissues compared to their matched non‐tumoral tissues (Figure 1c). The mRNA expression of PBRM1 was notably lower in 12 iCCA tissue specimens from patients treated at our center than in the matched adjacent non‐tumoral tissues (Figure 1d). Western blotting analysis of 8 samples yielded similar results for PBRM1 protein expression (Figure 1e). Immunohistochemical (IHC) staining using tissue microarray (TMA) was performed to assess PBRM1 expression in iCCA. A total of 185 iCCA patients were categorized into a PBRM1 high‐expression group (>4 points, *n* = 101, 54.6%) and a PBRM1 low‐expression group (≤4 points, *n* = 84, 45.4%) based on the IHC staining results (Figure 1f).

### 2.2 Low PBRM1 Expression Correlates with Aggressive Tumor Clinicopathological Characteristics and Poor Prognosis

Correlation analysis of PBRM1 protein levels with iCCA clinicopathologic features revealed a significant connection between PBRM1 down‐regulation and lymph node metastasis or advanced TNM stage (Table S2, Supporting Information).

Patients exhibiting high PBRM1 expression showed prolonged overall survival (OS) and disease‐free survival (DFS) compared to those with low PBRM1 expression (*p* = 0.026, *p* = 0.047, respectively, Figure 1g,h). The 1‐, 3‐, and 5‐year OS rates for patients with high PBRM1 expression were 48.8%, 25.1%, and 17.2%, respectively, significantly surpassing those for patients with low PBRM1 expression (37.6%, 13.4%, and 0%, respectively) (all, *p* < 0.05). Similar trends were observed in the 1‐, 3‐, and 5‐year DFS rates (34.5%, 20.5%, and 9.5% versus 22.8%, 11.5%, and 0%, respectively) (all, *p* < 0.05).

Multivariate analysis revealed that PBRM1 expression stood as an independent prognostic factor for OS and DFS in iCCA patients. Among other clinicopathological characteristics examined, only tumor differentiation and TNM stage significantly impacted the OS and DFS of iCCA patients Table S1, Supporting Information).

### 2.3 PBRM1 Suppresses iCCA Proliferation and Migration via the ERK1/2 Pathway In Vitro

**Figure 2:**
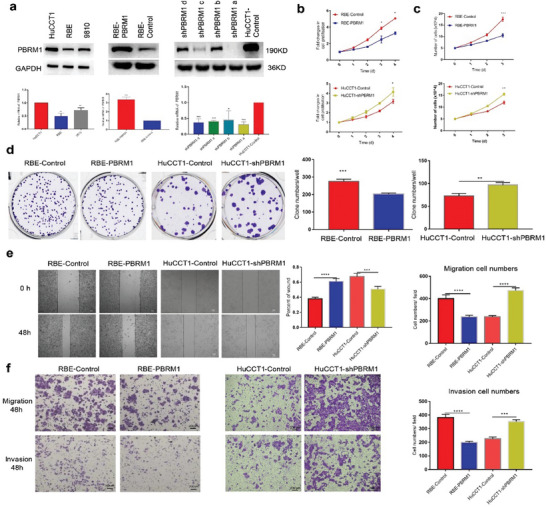
PBRM1 influenced the proliferation, migration, and invasion of iCCA in vitro. a) Selection and establishment of stably transfected iCCA cell lines with PBRM1 (RBE, HCCC 9810, HuCCT1), western blotting, and qRT‐PCR analyses showed successful overexpression PBRM1 of RBE cell lines and knockdown PBRM1 of HuCCT1 cell lines. b) The proliferative abilities of the stably transfected iCCA cells were determined by 3‐(4,5‐dimethylthiazol‐2‐yl)−2,5‐diphenyltetrazolium bromide assay, c) cell number counting, and d) plate cloning tests. e) Effects of PBRM1 overexpression and knockdown on migration using scratch wound healing assay and f) transwell migration assays. (f) The invasiveness of iCCA cells was determined by transwell invasion assays. ^*^ *p* < 0.05, ^**^ *p* < 0.01, ^***^ *p* < 0.001, ^****^ *p* < 0.0001.

To investigate the role of PBRM1 in relation to iCCA in vitro, we initially established stable PBRM1 overexpression and PBRM1 down‐expression iCCA cell lines using lentiviral vectors. The results indicated higher PBRM1 expression in the HuCCT1 cell line compared to the RBE and 9810 cell lines (**Figure** **2**a). The RBE cell line was transfected with a lentiviral vector carrying PBRM1 (RBE‐PBRM1) and its control (RBE‐Control). Four lentiviral short hairpin RNA (lenti‐shRNA) vectors were created to reduce PBRM1 expression in the HuCCT1 cell lines. ShPBRM1 a strongly decreased PBRM1, while shPBRM1 b, shPBRM1 c, and shPBRM1 d moderately decreased its expression (Figure 2a). Consequently, HuCCT1‐shPBRM1 was chosen for further experiments.

RBE‐PBRM1 displayed significantly reduced cell proliferation capabilities compared to RBE‐Control, while HuCCT1‐shPBRM1 exhibited notably increased cell proliferation capabilities compared to HuCCT1‐Control (Figure 2b). Cell counting tests yielded similar results (Figure 2c). These findings were further confirmed through plate cloning assays (Figure 2d). The cell scratch assay revealed that PBRM1 overexpression decreased RBE migration, whereas PBRM1 knockdown increased HuCCT1 migration (Figure 2e). Both Matrigel‐uncoated (migration) and Matrigel‐coated (invasion) Transwell assays demonstrated that PBRM1 overexpression in RBE significantly reduced migration and invasion, while PBRM1 knockdown in HuCCT1 enhanced cell migration and invasion (Figure 2f).

**Figure 3:**
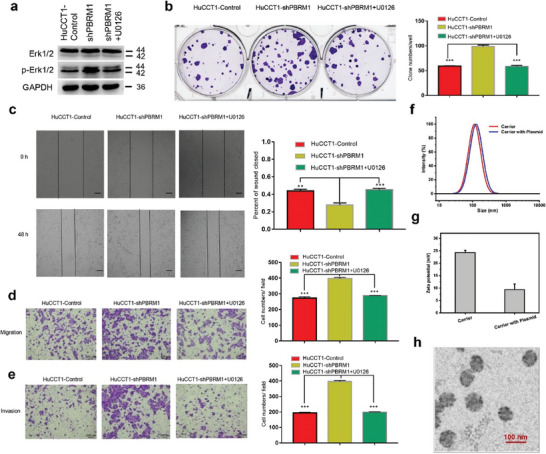
Down‐regulation of PBRM1 promoted proliferation, migration, and invasion of iCCA in vitro and a gene therapy nanomedicine has been developed to enhance expression of PBRM1. a) The protein levels of total and phosphorylation forms of ERK1/2 were compared in indicated cells. Effects of inhibitors of the REK1/2 pathway (U0126) on protein levels. Effects of inhibitors of the ERK1/2 pathway (U0126) on b) plate cloning tests, c) scratch wound healing assay, d) transwell migration assays, and e) transwell invasion assays. f) Particle size and g) zeta potential of carrier and carrier with PBRM1 plasmid. The zeta potential was represented as means ± S.D. (*n* = 5). h) Transmission electron microscopy (TEM) image of nanomedicine. Scale bar, 100 nm. ^*^ *p* < 0.05, ^**^ *p* < 0.01, ^***^ *p* < 0.001.

To preliminarily elucidate the regulatory pathway of PBRM1, HuCCT1‐shPBRM1 cells were treated with the ERK1/2 pathway inhibitor U0126. Following U0126 treatment, the phosphorylated protein p‐ERK1/2 levels in HuCCT1‐shPBRM1+ U0126 cells significantly decreased (**Figure** **3**a), indicating successful inhibition of ERK1/2 protein phosphorylation by U0126. The clone formation assay illustrated that specific inhibition of ERK1/2 activity by U0126 substantially hindered the proliferation of HuCCT1‐shPBRM1 cells (Figure 3b). The cell scratch test demonstrated reduced healing of PBRM1‐shPBRM1 cells by U0126 (Figure 3c). Moreover, U0126 notably impeded the migration and invasion of HuCCT1‐shPBRM1 cells, indicating that PBRM1 exerts an anti‐tumor effect through ERK1/2 pathway inhibition, a process blocked by an extracellular signal‐regulated kinase 1/2 (ERK1/2) inhibitor (Figure 3d–e).

### 2.4 A Reliable Gene Therapy Nanomedicine Was Developed Through the Design of Nanoparticles to Enhance PBRM1 Expression

The nanomedicines crafted in this research are intricate nanometer‐sized assemblies of cationic molecules paired with therapeutic nucleic acids. This fusion aims to uphold the stability and delivery efficiency of therapeutic plasmids during in vivo treatments, facilitating their optimal distribution within tumor tissues. Specifically, the nanomedicine formulated with PBRM1‐associated plasmids for DNA delivery has been meticulously characterized. The inclusion of multiple DNA molecules in the plasmid form ensures a consistent physical structure for both nanomedicine complexes. Through dynamic light scattering (DLS) assessments, it was ascertained that the nanocarriers' average diameter in aqueous solution measured ≈116 nm at room temperature, while the average diameter increased slightly to 120 nm upon plasmid incorporation (Figure 3f). Furthermore, the Zeta potential transitioned from +27 mV for the pure nanocarrier to +8 mV for the plasmid‐loaded nanocarrier (Figure 3g). Examination of TEM images revealed that each variant of nanomedicine exhibited a uniform spherical shape (Figure 3h). The reduced zeta potential and the TEM images substantiate the successful loading of plasmids onto the nanocarriers. With these findings, we are confident that the size and zeta potential of this nanomedicine is ideal for intravenous administration and intracellular endocytosis.

### 2.5 Nanomedicine‐Based Gene Therapy Has Superior Therapeutic Effect for In Vivo Cholangiocarcinoma Treatment Through Regulating the PBRM1 Expression

**Figure 4:**
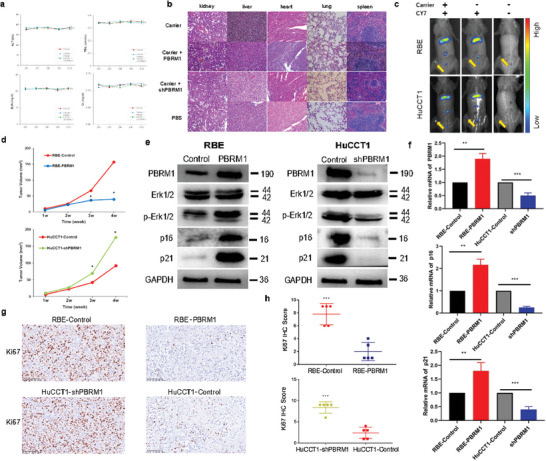
Nanomedicine has low toxicity, high tumor distribution, and a superior therapeutic effect for in vivo cholangiocarcinoma treatment. a) Serum liver function markers alanine transaminase (ALT)/total bilirubin (TBIL) and renal function markers blood urea nitrogen (BUN)/creatinine (Cr) were assessed during the period from 0 to 20 days after nanomedicine injection under therapeutic dosage. b) Haematoxylin and eosin (H&E) staining of the major organs from mice after 20 days of treatment for evaluations of tissue inflammation and structural damage. Scale bar, 20 µm. c) In vivo imaging was used to investigate the distribution of the nanomedicine using the near‐infrared fluorescent dye CY7 and injection from the tail vein, a significant concentration of fluorescence signal in the tumor area was observed. d) The tumor volume growth was obviously suppressed by PBRM1 overexpression plasmid‐loaded nanomedicine (up). The tumor volume growth was obviously accelerated by treatment with the shPBRM1 plasmid‐loaded nanomedicine (down). e) To determine whether the variations in tumor nodule growth were related to the changes in the expression change of PBRM1, the protein levels of PBRM1, p21, p16, ERK1/2, p‐ERK1/2 were compared in different nanomedicine groups, the mRNA levels of PBRM1, p21, and p16 were compared in different nanomedicine group. (f) Representative IHC staining of Ki67 in tumors from each group. (g) Statistical analysis of IHC staining of Ki67 in tumors from each group. ^*^ *p* < 0.05, ^**^ *p* < 0.01, ^***^ *p* < 0.001.

Following the intravenous administration of the PBRM1‐targeted nanomedicine, body biochemical data were meticulously analyzed. Serum biochemical assessments encompassed the liver functional clinical biomarkers alanine transaminase (ALT) and total bilirubin (TBIL), as well as the renal functional clinical biomarkers serum creatinine (Cr) and blood urea nitrogen (BUN). Noteworthy, no significant alterations in ALT, TBIL, BUN, and Cr levels were detected in animals receiving daily injections for 20 days (**Figure** **4**a). Additionally, histological examinations unveiled no apparent structural impairments, such as necrosis or cellular swelling, in vital organs post‐nanomedicine treatment over the same period (Figure 4b). These results underscored that the administration of the theranostic nanomedicine did not induce substantial adverse effects in vivo.

Subsequent staining of the nanomedicine with the near‐infrared fluorescent dye CY7 and injection via the tail vein revealed a marked accumulation of fluorescence signal in the tumor region. Importantly, this signal concentration in the tumor area was independent of CY7 itself (Figure 4c). These findings align with our previous study on nanomedicine distribution in tumors, underscoring the efficient delivery of plasmids to biliary tract cancer lesions, thereby facilitating in vivo tumor cell transfection.

To delve deeper into the tumor growth control function of nanomedicines in vivo, subcutaneous tumor models were established. Subsequently, mice were treated via tail vein injection with nanomedicines carrying PBRM1 overexpression plasmids and shPBRM1 plasmids. A control group received an equivalent volume of PBS. Notably, the tumor growth rate was markedly suppressed by the PBRM1 overexpression plasmid‐loaded nanomedicine, while it was significantly accelerated by the shPBRM1 plasmid‐loaded nanomedicine (Figure 4d).

To ascertain the relationship between tumor nodule growth variations and PBRM1 expression changes, western blotting assays were employed to assess PBRM1 expression levels. Results revealed a significant elevation in PBRM1, p21, and p16 expression in the RBE‐PBRM1 group compared to the RBE‐Control group, and a substantial decrease in the HuCCT1‐shPBRM1 group compared to the HuCCT1‐Control group (Figure 4e,f). Furthermore, immunohistochemistry (IHC) staining indicated that Ki67 expression was lowest in the RBE‐PBRM1 group (Figure 4g,h).

### 2.6 PBRM1 Inhibits iCCA Progression In Vivo

**Figure 5:**
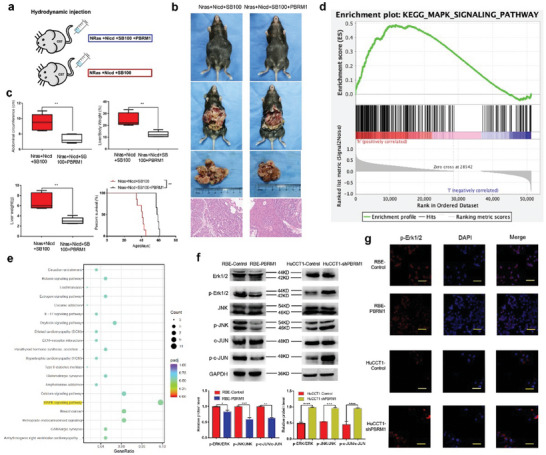
PBRM1 inhibited iCCA progression in vivo and down‐regulation of PBRM1 activates the ERK1/2 and JNK/c‐Jun signaling pathways. a) NRas/NICD‐induced iCCA formation with and without PBRM1 in the Hydrodynamic injection mouse model. b) Representative images of abdominal circumference values, liver and liver HE‐stained sections (*n* = 5). c) Box plots show the abdominal circumference values, liver weights, and liver‐to‐body weight ratios between group NRas/NICD+SB+PBRM1 and NRas/NICD+SB and Kaplan‐Meier curve of C57 mice injected with NRas/NICD+SB+PBRM1 or NRas/NICD+SB. d) GSEA enrichment analysis shows that PBRM1 is highly correlated with the MAPK signaling pathway. e) Bubble diagram of KEGG pathway analysis in RNA sequence of RBE‐PBRM1 versus RBE‐Control and HuCCT1‐Control versus HuCCT1‐shPBRM1 cells showed the top 20 enriched pathways. f) Protein levels of total and phosphorylation forms of ERK1/2, JNK, and c‐Jun were compared in stably transfected cells. g) Representative images of immunofluorescence staining of the indicated iCCA cell lines. ^*^ *p* < 0.05, ^**^ *p* < 0.01, ^***^ *p* < 0.001.

A hydrodynamic tail vein injection model with C57BL/6 mice was utilized to investigate whether PBRM1 can impede the tumor progression of iCCA cells in vivo (**Figure** **5**a). The findings revealed that the abdominal circumference, liver weight, and liver‐to‐body weight ratio of the PBRM1 group were notably lower than those of the control group (Figure 5b,c). Furthermore, the survival duration of the PBRM1 group surpassed that of the control group (Figure 5c). Gene Set Enrichment Analysis (GSEA) unveiled that PBRM1 expression in iCCA could influence various signaling pathways, including the common mitogen‐activated protein kinase (MAPK) and Wnt signaling pathways (FDR q‐val < 0.250) (Table S3, Supporting Information). These results suggest that the downregulation of PBRM1 in iCCA elevates the activity of the MAPK signaling pathway (Figure 5d).

Through mRNA sequencing analysis of RBE‐PBRM1 and RBE‐Control cells, along with HuCCT1‐Control and HuCCT11‐shPBRM1 cells, it was observed that among the top 20 enriched pathways, PBRM1 exhibited a strong correlation with the MAPK signaling pathway (Figure 5e). Western blotting was conducted to compare the levels of total proteins and phosphorylated proteins of ERK1/2 and JNK/c‐Jun in transfected iCCA cells. The expression of phosphorylated proteins in the ERK1/2 and JNK/c‐Jun pathways was markedly reduced in RBE‐PBRM1 cells when compared to RBE‐Control cells, whereas it was significantly elevated in HuCCT1‐shPBRM1 cells in contrast to HuCCT1‐Control cells (Figure 5f). This finding was further validated through an immunofluorescence experiment (Figure 5g). Thus, the PBRM1 expression level influences the activity of the MAPK pathway, collectively indicating that PBRM1 hinders iCCA progression in vivo.

### 2.7 PBRM1 Up‐Regulation Is Associated with Increased Genomic Instability and Inhibited iCCA Progression

**Figure 6:**
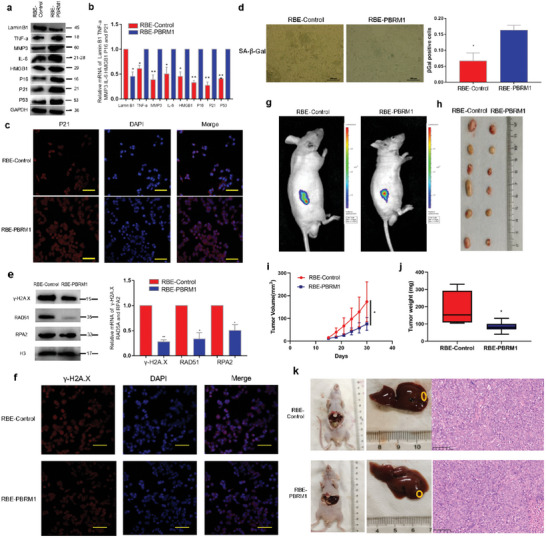
PBRM1 up‐regulation is associated with increased genomic instability and inhibited iCCA progression. a,b) Markers of senescence were detected by western blot in RBE‐Control and RBE‐PBRM1 cells. c) Representative images of immunofluorescence staining for γH2AX of the indicated iCCA cell lines. d) Cells imaged for SA‐β‐Gal and percentage of SA‐β‐Gal‐positive cells were determined for a minimum of 5 low‐power magnification fields in stably transfected cells. e) Protein levels of γH2AX, RAD51, and RPA2 foci were compared in stably transfected cells. f) Representative images of immunofluorescence staining for γH2AX of the indicated iCCA cell lines. g) Representative bioluminescence images of RBE‐Control and RBE‐PBRM1 group. h) Representative images of subcutaneous tumors derived from the indicated group. i) Tumor volume growth in mice subcutaneously injected with the indicated stable cell lines at indicated time points. j) Box plot showed the tumor weight growth between the RBE‐control and RBE‐PBRM1 groups (*n* = 6 mice per group). k) Representative images of nude mouse iCCA hepatic xenograft and HE‐stained sections. ^*^ *p* < 0.05, ^**^ *p* < 0.01, ^***^ *p* < 0.001.

Western blot analysis indicated that the upregulation of PBRM1 enhanced the expression of senescence‐associated secretory phenotype (SASP) markers, including HMGB1, IL‐6, TNF‐alpha, MMP3, and p16 (**Figure** **6**a). Moreover, the expression of Lamin B1 typically reduced in senescent cells, showed an inverse correlation with PBRM1 expression (Figure 6a). These findings were further supported by qPCR and immunofluorescence experiments (Figure 6b,c). SA‐β‐Gal staining was utilized to assess whether PBRM1 induced cellular senescence in iCCA cells, revealing a notable increase in SA‐β‐Gal‐positive cells upon PBRM1 upregulation (Figure 6d).

To explore the impact of PBRM1 on the expression of biomarkers associated with an ongoing DNA damage response (DDR), we evaluated the nuclear γH2AX foci in PBRM1‐overexpressing iCCA cells. In the absence of exogenous DNA damage, RBE‐PBRM1 cells exhibited a lower number of γH2AX foci compared to RBE‐Control cells (Figure 6e). This observation was further validated through an immunofluorescence experiment (Figure 6f).

Subcutaneous tumor models in nude mice and liver tumorigenicity models were established using RBE‐PBRM1 and RBE‐Control cells. Results demonstrated that tumors originating from the RBE‐Control group exhibited accelerated growth compared to those from the RBE‐PBRM1 group (Figure 6g,h). Tumor volume and weight were notably higher in the RBE‐Control group than in the RBE‐PBRM1 group (Figure 6i,j). Notably, significant differences were observed in hepatic xenograft tumors between the experimental and control groups (Figure 6k).

**Figure 7:**
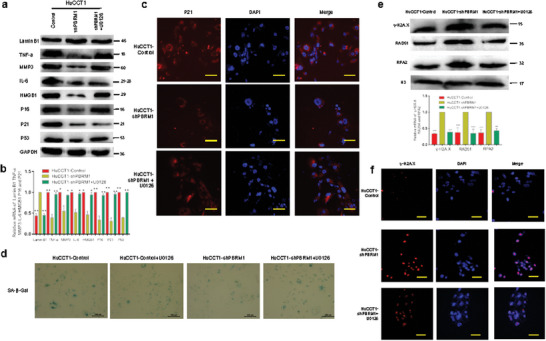
PBRM1‐ERK1/2 axis inhibited iCCA progression by cellular senescence induced by p16/p53/p21 and is reversed by ERK1/2 pathway inhibitor U0126 in vitro. a,b) Markers of senescence were detected by western blot in HuCCT1 cells. c) Representative images of immunofluorescence staining for P21 of the indicated iCCA cell lines. d) Effect of inhibitors of the REK1/2 pathway (U0126) on SA‐β‐Gal in indicated cells. e) Effects of inhibitors of the REK1/2 pathway (U0126) on protein levels of γH2AX, RAD51, and RPA2 foci in indicated cells. f) Representative images of immunofluorescence staining for γH2AX of the indicated iCCA cell lines by U0126. ^*^ *p* < 0.05, ^**^ *p* < 0.01, ^***^ *p* < 0.001.

To investigate the influence of ERK1/2 pathway activation on the senescence of HuCCT1‐shPBRM1 cells, we assessed the SASP markers p16, p21, and p53 in HuCCT1‐Control, HuCCT1‐shPBRM1, and HuCCT1‐shPBRM1+U0126 cells. Exposure to U0126 led to increased mRNA and protein expression of p16, p21, and p53 in HuCCT1‐shPBRM1 cells (**Figure** **7**a,b). Furthermore, qRT‐PCR and Western blotting of known SASP factors confirmed that inhibition of the ERK1/2 pathway significantly boosted the expression of HMGB1, IL‐6, TNF‐alpha, MMP3, and p16 in HuCCT1‐shPBRM1 cells following exposure to the ERK1/2 inhibitors U0126 (Figure 7a,b). These findings were further validated by immunofluorescence experiments (Figure 7c). SA‐β‐Gal staining indicated that ERK1/2 pathway inhibition led to a substantial increase in SA‐β‐Gal positivity in HuCCT1‐shPBRM1 cells treated with U0126 (Figure 7d). To explore the impact of ERK1/2 pathway activation on the genomic instability of HuCCT1‐shPBRM1 cells, we evaluated nuclear γH2AX, RAD51, and RPA2 foci in HuCCT1‐Control, HuCCT1‐shPBRM1, and HuCCT1‐shPBRM1+U0126 cells, revealing a decrease in the number of γH2AX, RAD51, and RPA2 foci after exposure to U0126 in HuCCT1‐shPBRM1 cells (Figure 7e,f).

### 2.8 PBRM1 Down‐Regulation Is Associated with Increased Genomic Instability and That Is Reversed by the ERK1/2 Pathway Inhibitor U0126

**Figure 8:**
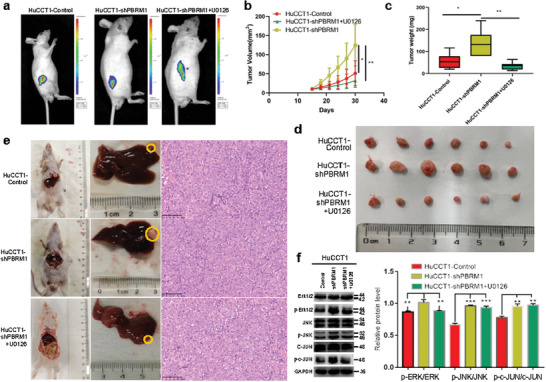
PBRM1 activates the ERK1/2 and JNK/c‐Jun signaling pathways inhibiting iCCA progression by cellular senescence and is reversed by ERK1/2 pathway inhibitor U0126 in vivo. a) Representative bioluminescence images of HuCCT1‐Control, HuCCT1‐shPBRM1, and HuCCT1‐shPBRM1+U0126 group. b) Tumor volume growth in mice subcutaneously injected with the indicated stable cell lines at indicated time points. c) Box plot showed the tumor weight growth between HuCCT1‐Control, HuCCT1‐shPBRM1, and HuCCT1‐shPBRM1+U0126 group. d) Representative images of subcutaneous tumors derived from the indicated group (*n* = 6 mice per group). e) Representative images of nude mouse iCCA hepatic xenograft and HE‐stained sections. f) The Protein levels of total and phosphorylation forms of ERK1/2, JNK, and c‐Jun were compared in tumor tissues. Effects of inhibitors of the ERK1/2 pathway (U0126) on protein levels. ^*^ *p* < 0.05, ^**^ *p* < 0.01, ^***^ *p* < 0.001.

Nude mice were used to establish subcutaneous tumor models and liver tumorigenicity models with HuCCT1‐Control, HuCCT1‐shPBRM1, and HuCCT1‐shPBRM1+U0126 cells. The results indicated that tumors originating from the HuCCT1‐shPBRM1 group exhibited faster growth compared to those from the HuCCT1‐Control group and the HuCCT1‐shPBRM1+U0126 group (**Figure** **8**a–e). Furthermore, tumor weight in the HuCCT1‐shPBRM1 group was higher than that in the HuCCT1‐Control group and the HuCCT1‐shPBRM1+U0126 group (Figure 8c). To validate the above pathway findings in vivo, the HuCCT1‐shPBRM1 group was treated with the ERK1/2 pathway inhibitor U0126. Following the U0126 treatment, the level of phosphorylated protein p‐ERK1/2 in tumor tissues of the HuCCT1‐shPBRM1+U0126 group significantly decreased (Figure 8f).

## 3 Discussion

In this study, we initially examined the mutations linked to cholangiocarcinoma using the TCGA dataset and identified PBRM1 mutation frequency at 20%, making it the most commonly mutated gene in iCCA. Despite this, limited reports exist on the function and precise molecular mechanism of PBRM1 in iCCA. Therefore, our research delved into exploring the role and potential mechanisms of PBRM1 in iCCA. Our findings revealed that reduced PBRM1 expression was associated with a poor prognosis. Multivariate analysis indicated that PBRM1 serves as an independent prognostic factor for OS and DFS in iCCA patients. Moreover, overexpression of PBRM1 was found to inhibit the proliferation, migration, and invasion of iCCA cells in vitro. Collectively, our results suggest that PBRM1 plays a tumor‐suppressive role in the onset and progression of iCCA.

Furthermore, our research unveiled a close connection between PBRM1 expression and the MAPK signal transduction pathway in iCCA through GSEA bioinformatics analysis and mRNA sequencing. The MAPK pathway can be activated by various extracellular stimuli, such as inflammatory factors, cytokines, and physical stimuli. Activation of the MAPK pathway is involved in key biological processes like cell proliferation, differentiation, and apoptosis.^[^ ^31^, ^32^, ^33^, ^34^, ^35^, ^36^, ^37^ ^]^ As an important member of MAPK, activation of ERK1/2 and JNK/c‐Jun signaling have been observed in a variety of cancers, including iCCA.^[^ ^38^, ^39^ ^]^ Previous studies confirmed that BAP1 deficiency increased the phosphorylation level of ERK1/2 and JNK/c‐Jun signal transduction, and thus promoted the progression of iCCA.^[^ ^40^ ^]^ Our study found that low expression of PBRM1 enhanced the phosphorylated protein expression levels of ERK1/2, JNK, and c‐Jun signaling pathways in iCCA cells, a finding that was reported for the first time. After phosphorylation, ERK protein can transfer from the cytoplasm to the nucleus to transmit signals.^[^ ^41^ ^]^ The addition of an ERK1/2 inhibitor showed that ERK1/2 phosphorylation induced by PBRM1 knockout is essential for iCCA cell proliferation, progression, and invasion. PBRM1 exerts an anti‐tumor effect by inhibiting the activity of the ERK1/2 and JNK/c‐Jun pathways, and the effect is blocked by ERK1/2 inhibition.

The phosphorylation of ERK1/2 and JNK/c‐Jun triggers the activation of several transcription factors, including essential downstream signaling molecules like c‐myc and cyclin D1, which regulate cell proliferation and growth.^[^ ^42^ ^]^ Aberrant activation of ERK1/2, JNK/c‐Jun, or their downstream targets have been identified as oncogenes that facilitate cell proliferation, and cell cycle progression, and enhance migration and invasion abilities of cancer cells.^[^ ^42^, ^43^ ^]^ A study showed that activation of the MAPK‐ERK pathway induced by YAP promotes glioma cells to enhance DNA damage repair and promotes the cell cycle, leading to tumor cell survival after radiotherapy.^[^ ^44^ ^]^ Interestingly, our study revealed that PBRM1 deficiency is linked to increased replication stress and genomic instability, both of which were alleviated by the ERK1/2 inhibitor U0126.

Cellular senescence is a physiological response to abnormal biological stress, either extracellular or intracellular. The tumor suppressor function of cellular senescence is well established because it blocks tumor cells at a certain stage to reduce cell proliferation. Cellular senescence can be identified by senescence biomarkers such as SA‐β‐Gal, SASP markers, and growth factors. The DNA damage response, chromatin remodeling, and SASP are highly associated with cellular senescence. It has also been widely demonstrated that AKT and ERK signaling is closely associated with cellular senescence.^[^ ^45^ ^]^ Down‐regulation of phosphorylated AKT and ERK levels causes apoptosis, cell cycle, and cellular senescence in several cancers including breast cancer.^[^ ^46^ ^]^ However, no study has shown that down‐regulation of phosphorylated MAPK‐ERK levels causes cellular senescence in iCCA. Additionally, there is no existing data indicating a close association between PBRM1 and cellular senescence. To our knowledge, our study for the first time identified that over‐expression of PBRM1 is associated with decreased phosphorylated ERK accumulation, and leads to cellular senescence. This novel finding underscores the need to further investigate the molecular mechanisms and signaling pathways involved in oncogene‐induced senescence (OIS).^[^ ^47^ ^]^

Almost all the OIS inducers trigger the activation of p53, induce the expression of its transcriptional target p21, and increase the expression of p16.^[^ ^48^ ^]^ p21 and p16 are downstream of pERK1/2 and are tumor suppressors that induce cellular senescence and cell cycle arrest.^[^ ^49^, ^50^, ^51^ ^]^ Studies have shown that inhibiting pERK1/2 or pAKT can activate p16 and p21, promoting cell senescence.^[^ ^52^, ^53^, ^54^ ^]^ Our research has uniquely demonstrated that inhibiting pERK1/2, which is upregulated by PBRM1 knockdown, can activate p53, p16, and p21, thereby inducing cell senescence in iCCA. Moreover, variations in IL‐6, TNF‐a, and MMP3 levels corresponded with p53 and p21 levels in iCCA. Additionally, the staining of SA‐β‐Gal and senescence‐associated proteins exhibited a positive correlation with the level of PBRM1.

It is widely known that nucleic acids cannot be directly treated in vivo through intravenous injection. Gene delivery through viral vectors carries a substantial risk of infection or immune‐related side effects.^[^ ^55^ ^]^ Therefore, we attempted to use a nanocarrier to achieve delivery of PBRM1, based on the experience gained in our nanocarrier's previous series of liver tumor studies.^[^ ^56^, ^57^, ^58^ ^]^ Through delivery tracing assays, we confirmed that these nanocarriers could enhance the intra‐tumor distribution of plasmids via fluorescence imaging experiments. This successful transfection of tumor cells in vivo using nanocarriers offers promise for leveraging therapeutic genes in cholangiocarcinoma therapy. Our in vivo experiments validated that nanomedicines can precisely modulate PBRM1 expression in cholangiocarcinoma lesions, thereby exerting negative control over subcutaneous tumor growth. This breakthrough signifies the successful implementation of in vivo gene therapy for cholangiocarcinoma using nanomedicine.

In conclusion, our study highlights that PBRM1 not only regulates iCCA progression but also independently predicts iCCA prognosis. We have successfully developed a reliable gene therapy nanomedicine for in vivo cholangiocarcinoma treatment through modulating PBRM1 expression. Targeting the PBRM1‐ERK1/2‐cellular senescence axis with U0126 holds promising clinical potential in iCCA treatment.

## Conflict of Interest

The authors declare no conflict of interest.

## Author Contributions

X.W.W., Y.Z., Y.D., and J.L.Y. contributed equally to this work as the first author. S.L.S and J.W. lead and supervise the project, funding support, edit, and revise the manuscript. S.L.S., J.W., and X.W.W. conceived the research and designed the experiments. X.W.W., Y.Z., and J.L.Y. carried out the animal experiments and analyzed the data. Y.D. performed the development of nanomedicine and analyzed the data. Y.Z. provided clinical samples and performed histopathological assessments. Z.M.S. and S.R.L. provided suggestions and technical support in experiments. R.H.Z. helped with manuscript preparation. X.W.W. and Y.D. wrote the manuscript. All authors discussed the results and commented on the manuscript.

## Supporting information



Supporting Information

## Data Availability

The raw sequencing data analyzed in the current study is available from the corresponding author on reasonable request.
