# Supplementary material for: Nanosize Non‐Viral Gene Therapy Reverses Senescence Reprograming Driven by PBRM1 Deficiency to Suppress iCCA Progression
Source: Adv Sci (Weinh). 2025 Jan 17;12(10):2414525. doi: 10.1002/advs.202414525 (PMC11904949; doi:10.1002/advs.202414525)
Supplement: Supplementary file 1 — Supporting Information [file ADVS-12-2414525-s001.pdf]

ADVANCED SCIENCE
Open Access

# Supporting Information

for *Adv. Sci.*, DOI 10.1002/advs.202414525

Nanosize Non-Viral Gene Therapy Reverses Senescence Reprograming Driven by PBRM1 Deficiency to Suppress iCCA Progression

*Xiwen Wu, Yi Zhang, Yuan Ding, Jiali Yang, Zimin Song, Shuirong Lin, Ruhe Zhang, Jun Wu* and Shunli Shen**

*Supplemental information for*

**Nanosize non-viral gene therapy reverses senescence reprograming driven by PBRM1 deficiency to suppress iCCA progression**

**Authors: Xiwen Wu[1,2,†], Yi Zhang[1,3†], Yuan Ding[1,†], Jiali Yang[1,†], Zimin Song[1], Shuirong Lin[1], Ruhe Zhang[4],** Jun Wu[5,6,*], Shunli Shen[1,*]

**Affiliations:**

[1]Department of Hepatic Surgery, Center of Hepato-Pancreato-Biliary Surgery, The First Affiliated Hospital of Sun Yat-sen University, Guangzhou, Guangdong, 510080, China;

[2]Department of Clinical Nutrition, Sun Yat-sen University Cancer Center; State Key Laboratory of Oncology in South China; Guangdong Provincial Clinical Research Center for Cancer; Collaborative Innovation Center for Cancer Medicine, Guangzhou 510060, China

[3]Department of Hepatobiliary Surgery, The Third Affiliated Hospital of Sun Yat-sen University, Guangzhou, 510630, China

[4] Department of Hematology, The Seventh Affiliated Hospital of Sun Yat-sen University, Shenzhen, 518107, China

[5]Bioscience and Biomedical Engineering Thrust, The Hong Kong University of Science and Technology (Guangzhou), Nansha, Guangzhou, 511400, Guangdong, China

[6]Division of Life Science, The Hong Kong University of Science and Technology, Hong Kong SAR, 999077, China

[†] These authors contribution equally to this work.

***Corresponding authors:** Shunli Shen

Email: shenshli@mail.sysu.edu.cn;

**Co-correspondence Authors:** Jun Wu

Email: junwuhkust@ust.hk;

# Experimental Section

## Material and Methods

### *Patients and follow-up*

From 2004 to 2015, a total of 185 iCCA patients from the First Affiliated Hospital of Sun Yat-Sen University were included in the study. Prior informed consent was obtained from each participant, and the research was approved by the ethics committee of Sun Yat-sen University. Patients underwent follow-up evaluations every 3 months in the first year after operation, followed by assessments every 6 months thereafter. The follow-up period concluded in August 2016, with an average follow-up duration of 13.6 months (ranging from 2 to 50 months). All tumor samples were pathologically confirmed as iCCA. Disease-free survival (DFS) duration was defined as the interval from the operation to recurrence, while overall survival (OS) duration was defined as the time from the operation to iCCA-related mortality.

### *Tissue microarrays (TMA) construction and immunohistochemistry*

Tissue microarrays (TMA) and immunohistochemistry were constructed and performed as previously described (1). Rabbit polyclonal anti-human PBRM1 antibodies (1:100, Abcam, USA) were used for IHC according to the reagent instructions. The results of IHC staining were determined by the immunoreactivity scores (IRSs). The IRSs were calculated as described in our previous study and were independently judged by two researchers (2).

***Cell lines and lentiviral transfection***

Three human iCCA cell lines, RBE, HuCCT1 and HCCC-9810 were procured from the Guangzhou Cellcook Biotech Co., Ltd (Guangzhou, China). iCCA cells were cultured in RPMI-1640 (Gibco, USA) supplemented with 10% fetal bovine serum (FBS) (Gibco, USA) and 1% penicillin/streptomycin (Gibco, USA). All cell lines were maintained at 37°C in an environment with 5% $CO_2$. The lentiviral vectors with PBRM1 overexpression were transfected into RBE cells and designated as RBE-PBRM1 cells, while the empty lentiviral vectors (GeneCopeia, Guangzhou, China) were also transfected into RBE cells and designated as RBE-Control cells. The short hairpin PBRM1 lentiviral vectors (GeneCopeia, Guangzhou, China) designated for knockdown of PBRM1 were transfected into HuCCT1 cells, while the empty lentiviral vectors were also transfected into HuCCT1 cells and designated as HuCCT1-Control cells. The target sequences of PBRM1 overexpression and knockdown plasmid were shown in Supplementary Table S4. Combined with previous literature and pre-experiments, the dose and time of U0126 (Cell Signaling Technology, CST) on HuCCT1-shPBRM1 cells were 10 μ M and 1 hour. All the lentiviral vectors were conducted with enhanced green fluorescent protein gene and puromycin resistance sequences.

***Therapeutic gene loading by nanocarriers and characterization of nanomedicine***

Nanocarrier *Entranster$^{TM}$-in vivo* Transfection Reagent (PAMAM, Engreen Biosystem, Auckland, New Zealand) was used for the *in vivo* PBRM1 overexpression plasmids, shPBRM1 plasmids or negative control plasmids transfection through intravenous

injection into the mouse tail vein according to the manufacturer's instructions (3). We prepared a nucleic acid dilution by dissolving 30 μg plasmids in 20 μl PBS. Then, we added 20 μL PBS and mixed the solution well. Next, we prepared a transfection reagent dilution by dissolving 20 μL diluted transfection reagent in 20 μL PBS, and the solution was mixed well. Finally, the plasmids' dilution and transfection reagent dilution were mixed, to form nanomedicines carrying the corresponding plasmids, for *in vivo* intravenous injection (4).

The hydrodynamic size and zeta potential of nanocarriers and nanocarriers with plasmid were determined by dynamic light scattering (DLS) method. Zeta potential measurements of nanocarriers were performed in standard capillary electrophoresis cells, using a ZetaPlus instrument (Brookhaven, USA), at a 45° angle at 25°C. The mean plus standard deviation is based on data from five runs. The size of the nanocarriers was measured at 25°C on the same instrument and the scattered light is detected at a 90° angle. The morphology of nanomedicine was observed with transmission electron microscopy (TEM; JEOL 2000FX, Japan) operated at 120 kV. Nanomedicine solution with a concentration of 0.5 mg/mL was prepared and 5μL of solution was dropped on a copper net. After the solution dries, TEM observation can be performed.

***Animal studies and HE staining***

All animal studies were following the Guide for the Care and Use of Laboratory Animals (NIH publication nos. 80-23, revised 1996) and were approved by the

Institutional Animal Care and Use Committee of Sun Yat-sen University (Guangzhou, China, 2020-0076, 2020-0273). Male BALB/c-Nude mice (4 weeks old) and C57BL/6 mice (4 weeks old) were procured from GemPharmatech Co., Ltd and housed in the animal experiment center of Sun Yat-sen University. RBE-PBRM1, RBE-Control, HuCCT1-shPBRM1, and HuCCT1-Control cells ($1\times 10^7$) were individually suspended in 100 μL of RPMI-1640 medium with 100 μL of Matrigel (BD Biosciences, USA) and then subcutaneously injected into the flanks of BALB/c-Nude mice. Four weeks post-implantation, the fluorescence distribution in the mice was assessed using an in vivo imaging system (IVIS, PerkinElmer). Tumor dimensions were measured biweekly, and the volume was calculated using the formula: $V= \text{length} \times \text{width}^2/2$. After euthanasia, the tumors were removed, weighed and photographed according to different group 4 weeks later. Then the xenografts were cut into $1 \times 1 \times 1\ mm^3$ piece and the tumor piece was surgically implanted in the largest liver lobe of the BALB/c-Nude mice (4 weeks old, 5 per group) which were anesthetized by 2% Pentobarbital Sodium (45mg/kg). All surgical operations were performed in 30 minutes on a sterile clean table in the SPF operating room. Two weeks after inoculation of HuCCT1-Control cells and HuCCT1-shPBRM1 cells, 25 μM/kg U0126 were injected intraperitoneally three times a week to inhibit ERK1/2 signal pathway.

We conducted hydrodynamic tail (HDT) vein injection model in C57BL/6 mice to verify whether PBRM1 can inhibit the tumor progression of iCCA cells in vivo. We divided the C57BL/6 female mice randomly into two groups, each consisting of 12 mice. PBRM1 group: 20ug NRAS plasmid + 20ug NICD plasmid + 1.6ug sleeping

beauty (SB) +20ug PBRM1 plasmid, Control group: 20 ug NRAS plasmid + 20 ug NICD plasmid + 1.6ug SB. All plasmids were donated by Professor Xuxu Sun of Shanghai Jiao Tong University. Five weeks after successful HDT modeling, 5 mice in each group were sacrificed, and the data of abdominal circumference, liver weight and mouse body weight were measured. The survival time of the remaining 7 mice of two groups was recorded.

***In vivo toxicity assay.***

To measure the side effects of nanomedicine, mice-bearing tumor were divided randomly into 3 groups (n = 6 per group). Animals received a single injection of 150 μL neat PBS (PBS group), the same volume of PBS containing nanocarriers (carrier group), 150 μL PBS containing nanocarriers with PBRM1 overexpression plasmid dose of 8 μg (carrier+PBRM1 group), and 150 μL PBS containing nanocarriers with shPBRM1 plasmid dose of 8 μg (carrier+shPBRM1 group) via the tail vein. To determine the state of the liver and renal, serum biochemical tests for liver function biomarkers ALT or TBIL and renal function biomarkers BUN or Cr were performed over 72 h after the injection. To further determine the long-term solid organ injury, kidney, liver, heart, lung and spleen tissues harvested from mice of carrier+PBRM1, carrier+shPBRM1 or PBS groups after 20 days of treatment were subjected to the H&E staining.

***Nanomedicine in vivo delivery tracing assays***

*In vivo* nanomedicine delivery tracing assays were performed after the implantation tumor of control group grew up to 100 mm$^3$. In order to observe the *in vivo* nanomedicine delivery into tumor, we used the near-infrared fluorescent (NIRF) dye CY7 to label the nanocarrier before injection (5). *In vivo* NIRF fluorescence scanning was performed 2h after injection as previous reported (2).

***Bioinformatics analysis and mRNA sequence***

Bioinformatics is a common data processing tool for microarray RNA database and we conducted gene set enrichment analysis (GSEA) analysis. We download the mRNA data of 36 iCCA patients in the format of Htseq-count from the Cancer Genome Atlas (TCGA) (https://cancergenome.nih.gov/) database. 36 iCCA samples were divided into high expression group and low expression group by the median expression of PBRM1 and made into phenotypic data files (csl format, divided into PBRM1 high and PBRM1 low). Then, we used gene set enrichment analysis (GSEA) software (version 3.0) provided by GSEA official website (http://software.broadinstitute.org/gsea/index.jsp). RBE-PBRM1 and RBE-Control cells, HuCCT1-Control and HuCCT1-shPBRM1 cells were sent for mRNA sequence seeking differentially expressed genes and enriched pathways. The bubble chart of KEGG pathway showed the first 20 enriched pathways.

***Xenografted tumor model and in vivo treatment with nanomedicines***

As described in the previous section, the immunodeficient mice were randomly divided into groups (n = 6 per group). RBE cells or HuCCT1 cells were subcutaneously injected

into the dorsal left flank of nude mice. For each mouse, we injected 100 μl nanomedicine working solution for each group through the tail vein once every 3 days. Tumors were measured twice a week. On day 28, the tumors were harvested and fresh tumor tissue was used for the western blot assays (6).

***Cell proliferation, Cell counting, plate cloning, scratching, Matrigel migration and invasion, western blot and q RT-PCR***

Cell proliferation, cell counting and plate cloning were performed as described previously (7). Scratch, Matrigel migration and invasion were also performed as described previously (7). The proteins extracted from iCCA cells and fresh tumor tissues/normal tissues were compared by western blot. Total RNA and Quantitative Real-Time PCR (qRT-PCR) were performed as described previously (7). The sequences of the gene-specific primers for PBRM1 were: PBRM1 Forward Primer, 5'-GCGGTAGGCGTGTACGGT-3'; PBRM1 Reverse Primer, 5'-CTGGAATAGCTCAGAGGC-3'. GAPDH forward, 5′-AGCCACATCGCTCAGACAC-3′; GAPDH reverse, 5′-GAATTTGCCATGGGTGGA-3′.

***Immunofluorescence***

Cells were fixed with 4% paraformaldehyde for 15 minutes and washed three times with PBS. Primary antibodies against p-ERK1/2 (Cell Signaling Technology), γH2AX (Cell Signaling Technology) and P21 (Cell Signaling Technology) were incubated

overnight at 4°C. DAPI was utilized as a nuclear counterstain (Genecopia, Guangzhou, China). Images were captured using a Leica DMRA fluorescence microscope (Leica, Wetzlar, Germany).

***SA-β-Gal staining***

Cells were fixed and stained for the SA-β-Gal according to the manufacturer's instructions (Senescence β-Galactosidase Staining Kit, Beyotime, China). The manual calculation was employed to determine the positive ratio of SA-β-Gal-positive cells.

***Statistical analysis***

All experiments were repeated three times. The measurement data were presented as mean ± standard deviation. The Student's t-test was employed for intergroup comparisons, while the chi-square test was used for clinicopathological feature analysis. Survival analysis was carried out using the log-rank test and Kaplan-Meier analysis. Multivariate survival analysis was performed using the Cox proportional hazards model. Statistical analysis was conducted using SPSS v20.0 software for Windows (IBM, Chicago, IL, USA), with significance set at $P < 0.05$ (two-tailed).

**Table S1 Univariate and multivariate analysis of prognostic factors in iCCA patients (n=185)**

| Category | Overall Survival (OS) | | | Disease-Free Survival (DFS) | | |
|---|---|---|---|---|---|---|
| | Univariate | Multivariate | | Univariate | Multivariate | |
| | ***P*** | **HR (95% CI)** | ***P*** | ***P*** | **HR (95% CI)** | ***p*** |
| Age (>60 vs. ≤60 years) | 0.693 | | NA | 0.55 | | NA |
| Cirrhosis (No vs. Yes) | 0.072 | 1.49(1.00-2.21) | 0.05 | 0.042 | 1.55 (1.06-2.27) | 0.03 |
| Tumor size (cm) (≤5 vs. >5) | 0.002 | 1.52(1.05-2.19) | 0.03 | <0.001 | | NS |
| Number (Single vs. Multiple) | 0.006 | 1.52(1.06-2.17) | 0.02 | 0.002 | | NS |
| Differentiation (W+M vs. P) | 0.001 | 1.89(1.32-2.72) | <0.001 | 0.024 | 1.44( 1.00-2.05) | 0.05 |
| Bloodloss (≤400 ml vs.>400 ml) | 0.029 | | NS | 0.009 | | NS |
| Distant metastasis (No vs. Yes) | 0.003 | | NS | 0.003 | | NS |
| TNM (I+II vs. III+IV) | <0.001 | 2.19(1.55-3.11) | <0.001 | <0.001 | 1.80(1.27-2.54) | <0.001 |
| Resection margin (R0 vs. R1) | 0.001 | | NS | 0.002 | | NS |
| Lymph node metastasis (No vs. Yes) | <0.001 | | NS | <0.001 | | NS |
| CA19-9 (IU/mL) (≤35 *vs*. >35) | 0.265 | | NA | 0.397 | | NS |
| PBRM1 (Low *vs*. High) | 0.026 | 0.72(0.52-1.00) | 0.05 | 0.047 | 0.72(0.52-1.00) | 0.05 |

Abbreviations **iCCA**, Intrahepatic cholangiocarcinoma**, HBsAg**, hepatitis B surface antigen, **W+M**, well + moderated differentiation, **P**, poor differentiation, **TNM**, Tumor Node Metastasis, **CEA**, Carcinoembryonic antigen, **CA19-9**, carbohydrate antigen 19-9, **PBRM1,** polybromo 1, **NA**, not applicable, **NS**, not significant.

**Table S2 Correlation between PBRM1 expression and clinicopathologic features of iCCA (n = 185)**

| Category Subcategory | NO. | PBRM1 High (n=*101*) | PBRM1 Low (n=*84*) | *p*.overall |
|---|---|---|---|---|
| Gender | | | | 0.894 |
| Female | 86 | 46 (45.5%) | 40 (47.6%) | |
| Male | 99 | 55 (54.5%) | 44 (52.4%) | |
| age: | | | | 1 |
| ≤60 | 114 | 62 (61.4%) | 52 (61.9%) | |
| >60 | 71 | 39 (38.6%) | 32 (38.1%) | |
| bloodloss: | | | | 0.462 |
| ≤400 ml | 110 | 63 (62.4%) | 47 (56.0%) | |
| >400 ml | 75 | 38 (37.6%) | 37 (44.0%) | |
| blood transfusion: | | | | 0.367 |
| No | 122 | 70 (69.3%) | 52 (61.9%) | |
| Yes | 63 | 31 (30.7%) | 32 (38.1%) | |
| cirrhosis: | | | | 0.614 |
| No | 143 | 80 (79.2%) | 63 (75.0%) | |
| Yes | 42 | 21 (20.8%) | 21 (25.0%) | |
| numbers: | | | | 1 |
| Single | 127 | 69 (68.3%) | 58 (69.0%) | |
| Multiple | 58 | 32 (31.7%) | 26 (31.0%) | |
| diameter: | | | | 0.333 |
| ≤5 cm | 69 | 34 (33.7%) | 35 (41.7%) | |
| >5 cm | 116 | 67 (66.3%) | 49 (58.3%) | |
| differentiation[a]: | | | | 0.52 |
| well+moderated | 131 | 74 (73.3%) | 57 (67.9%) | |
| poor | 54 | 27 (26.7%) | 27 (32.1%) | |
| Lymphnode metastasis: | | | | 0.045 |
| No | 112 | 54 (53.5%) | 58 (69.0%) | |
| Yes | 73 | 47 (46.5%) | 26 (31.0%) | |
| distant metastasis: | | | | 0.17 |
| No | 147 | 76 (75.2%) | 71 (84.5%) | |
| Yes | 38 | 25 (24.8%) | 13 (15.5%) | |
| Resection margin: | | | | 0.181 |
| R1 | 77 | 47 (46.5%) | 30 (35.7%) | |
| R0 | 108 | 54 (53.5%) | 54 (64.3%) | |
| TNM[b]: | | | | 0.034 |
| I+II | 78 | 35 (34.7%) | 43 (51.2%) | |
| III+IV | 107 | 66 (65.3%) | 41 (48.8%) | |
| CEA: | | | | 0.64 |
| ≤5.0 IU/L | 110 | 58 (57.4%) | 52 (61.9%) | |
| >5.0 IU/L | 75 | 43 (42.6%) | 32 (38.1%) | |
| CA199: | | | | 0.576 |
| ≤35 IU/L | 69 | 40 (39.6%) | 29 (34.5%) | |

| | | | |
|---|---|---|---|
| >35 IU/L | 116 | 61 (60.4%) | 55 (65.5%) |

Abbreviations: **PBRM1,** polybromo 1**, iCCA**, Intrahepatic cholangiocarcinoma**, HBsAg**, hepatitis B surface antigen, **TNM**, Tumor Node Metastasis, **CEA,** Carcinoembryonic antigen, **CA19-9**, carbohydrate antigen 19-9.

[a] According to the World Health Organization (WHO) classification of tumors of the digestive system 2010,

[b] Based on seventh edition cancer staging manual of American Joint Committee on Cancer.

**Table S3 Enrichment analysis of the effect of PBRM1 expression changes on related signaling pathways in iCCA**

| No. | NAME | ES | NES | NOM p-val | FDR q-val |
|---|---|---|---|---|---|
| 1 | KEGG_ARRHYTHMOGENIC_RIGHT_VENTRICULAR_CARDIOMYOPATHY_ARVC | 0.6282 | 1.7194 | 0.00418 | 0.332276 |
| 2 | KEGG_DILATED_CARDIOMYOPATHY | 0.57646 | 1.6931 | 0.0042 | 0.243433 |
| 3 | KEGG_HEDGEHOG_SIGNALING_PATHWAY | 0.59395 | 1.6921 | 0.00405 | 0.164319 |
| 4 | KEGG_WNT_SIGNALING_PATHWAY | 0.53776 | 1.6918 | 0 | 0.124491 |
| 5 | KEGG_ENDOCYTOSIS | 0.49633 | 1.6821 | 0 | 0.110686 |
| 6 | KEGG_TGF_BETA_SIGNALING_PATHWAY | 0.57338 | 1.655 | 0.00658 | 0.127223 |
| 7 | KEGG_FC_GAMMA_R_MEDIATED_PHAGOCYTOSIS | 0.5583 | 1.6272 | 0.00394 | 0.150667 |
| 8 | KEGG_MAPK_SIGNALING_PATHWAY | 0.48744 | 1.622 | 0.00217 | 0.140886 |
| 9 | KEGG_ADHERENS_JUNCTION | 0.56926 | 1.6202 | 0.00433 | 0.130007 |
| 10 | KEGG_HYPERTROPHIC_CARDIOMYOPATHY_HCM | 0.56769 | 1.6202 | 0.0131 | 0.117006 |
| 11 | KEGG_BASAL_CELL_CARCINOMA | 0.57978 | 1.6151 | 0 | 0.112989 |
| 12 | KEGG_NOTCH_SIGNALING_PATHWAY | 0.55411 | 1.6011 | 0.01411 | 0.121003 |
| 13 | KEGG_VASCULAR_SMOOTH_MUSCLE_CONTRACTION | 0.52119 | 1.5993 | 0.0061 | 0.113507 |
| 14 | KEGG_PATHWAYS_IN_CANCER | 0.4777 | 1.593 | 0.00211 | 0.114635 |
| 15 | KEGG_PROSTATE_CANCER | 0.4829 | 1.5919 | 0.00406 | 0.108233 |
| 16 | KEGG_INOSITOL_PHOSPHATE_METABOLISM | 0.54288 | 1.5866 | 0.00423 | 0.106153 |
| 17 | KEGG_AXON_GUIDANCE | 0.5327 | 1.5819 | 0.0046 | 0.105368 |
| 18 | KEGG_LONG_TERM_POTENTIATION | 0.54261 | 1.5688 | 0.01037 | 0.113218 |
| 19 | KEGG_PHOSPHATIDYLINOSITOL_SIGNALING_SYSTEM | 0.54976 | 1.5683 | 0.01616 | 0.107604 |
| 20 | KEGG_SMALL_CELL_LUNG_CANCER | 0.51104 | 1.557 | 0.02045 | 0.114259 |

Abbreviations: **ES**, Enrichment Score, **NES**, Normalized Enrichment Score, **Nom P-value**, Nominal p-value, **FDR q-value**, False Discovery Rate q-value. FDR q-value < 0.250 be considered as different.

**Table S4 PBRM1 overexpression plasmid sequences (ORF sequence information)**

>EX-H1992-Lv201 ORF sequence

ATGGGTTCCAAGAGAAGAAGAGCTACCTCCCCTTCCAGCAGTGTCAGCGGGGACTTTGATGATGGGCACCATTCT
GTGTCAACACCAGGCCCAAGCAGGAAAAGGAGGAGACTTTCCAATCTTCCAACTGTAGATCCTATTGCCGTGTGC
CATGAACTCTATAATACCATCCGAGACTATAAGGATGAACAGGGCAGACTTCTCTGTGAGCTCTTCATTAGGGCAC
CAAAGCGAAGAAATCAACCAGACTATTATGAAGTGGTTTCTCAGCCCATTGACTTGATGAAAATCCAACAGAAAC
TAAAAATGGAAGAGTATGATGATGTTAATTTGCTGACTGCTGACTTCCAGCTTCTTTTTAACAATGCAAAGTCCTA
TTATAAGCCAGATTCTCCTGAATATAAAGCCGCTTGCAAACTCTGGGATTTGTACCTTCGAACAAGAAATGAGTTT
GTTCAGAAAGGAGAAGCAGATGACGAAGATGATGATGAAGATGGGCAAGACAATCAGGGCACAGTGACTGAAGG
ATCTTCTCCAGCTTACTTGAAGGAGATCCTGGAGCAGCTTCTTGAAGCCATAGTTGTAGCTACAAATCCATCAGGA
CGTCTCATTAGCGAACTTTTTCAGAAACTGCCTTCTAAAGTGCAATATCCAGATTATTATGCAATAATTAAGGAGC
CTATAGATCTCAAGACCATTGCCCAGAGGATACAGAATGGAAGCTACAAAAGTATTCATGCAATGGCCAAAGATA
TAGATCTCCTCGCAAAAAATGCCAAAACTTATAATGAGCCTGGCTCTCAAGTATTCAAGGATGCAAATTCAATTAA
AAAAATATTTTATATGAAAAAGGCTGAAATTGAACATCATGAAATGGCTAAGTCAAGTCTTCGAATGAGGACTCC
ATCCAACTTGGCTGCAGCCAGACTGACAGGTCCTTCACACAGTAAAGGCAGCCTTGGTGAAGAGAGAAATCCCAC
TAGCAAGTATTACCGTAATAAAAGAGCAGTACAAGGAGGTCGTTTATCAGCAATTACAATGGCACTTCAATATGG
CTCAGAAAGTGAAGAAGATGCTGCTTTAGCTGCTGCACGCTATGAAGAGGGAGAGTCAGAAGCAGAAAGCATCA
CTTCCTTTATGGATGTTTCAAATCCTTTTTATCAGCTTTATGACACAGTTAGGAGTTGTCGGAATAACCAAGGGCAG
CTAATAGCTGAACCTTTTTACCATTTGCCTTCAAAGAAAAAATACCCTGATTATTACCAGCAAATTAAAATGCCCA
TATCACTACAACAGATCCGAACAAAACTGAAGAATCAAGAATATGAAACTTTAGATCATTTGGAGTGTGATCTGA
ATTTAATGTTTGAAAATGCCAAACGCTATAATGTGCCCAATTCAGCCATCTACAAGCGAGTTCTAAAATTGCAGCA
AGTTATGCAGGCAAAGAAGAAAGAGCTTGCCAGGAGAGACGATATCGAGGACGGAGACAGCATGATCTCTTCAG
CCACCTCTGATACTGGTAGTGCCAAAAGAAAAAGTAAAAAGAACATAAGAAAGCAGCGAATGAAAATCTTATTCA
ATGTTGTTCTTGAAGCTCGAGAGCCAGGTTCAGGCAGAAGACTTTGTGACCTATTTATGGTTAAACCATCCAAAAA
GGACTATCCTGATTATTATAAAATCATCTTGGAACCAATGGACTTGAAAATAATTGAGCATAACATCCGCAATGAC
AAATATGCTGGTGAAGAGGGAATGATAGAAGACATGAAGCTGATGTTCCGGAATGCCAGGCACTATAATGAGGA
GGGCTCCCAGGTTTATAATGATGCACATATCCTGGAGAAGTTACTCAAGGAGAAAAGGAAAGAGCTGGGCCCACT
GCCTGATGATGATGACATGGCTTCTCCCAAACTCAAGCTGAGTAGGAAGAGTGGCATTTCTCCTAAAAAATCAAA
ATACATGACTCCAATGCAGCAGAAACTAAATGAGGTCTATGAAGCTGTAAAGAACTATACTGATAAGAGGGGTCG
CCGCCTCAGTGCCATATTTCTGAGGCTTCCCTCTAGATCTGAGTTGCCTGACTACTATCTGACTATTAAAAAGCCCA
TGGACATGGAAAAAATTCGAAGTCACATGATGGCCAACAAGTACCAAGATATTGACTCTATGGTTGAGGACTTTG
TCATGATGTTTAATAATGCCTGTACGTACAATGAGCCGGAGTCTTTGATCTACAAAGATGCTCTTGTTCTACACAA
AGTCCTGCTTGAAACACGCAGAGACCTGGAGGGAGATGAGGACTCTCATGTCCCAAATGTGACTTTGCTGATTCA
AGAGCTTATCCACAATCTTTTTGTGTCAGTCATGAGTCATCAGGATGATGAGGGAAGATGCTACAGCGATTCTTTA
GCAGAAATTCCTGCTGTGGATCCCAACTTTCCTAACAAACCACCCCTTACATTTGACATAATTAGGAAGAATGTTG
AAAATAATCGCTACCGTCGGCTCGATTTATTTCAAGAGCATATGTTTGAAGTATTGGAACGAGCAAGAAGGATGA
ATCGGACAGATTCAGAAATATATGAAGATGCAGTAGAACTTCAGCAGTTTTTTATTAAAATTCGTGATGAACTCTG
CAAAAATGGAGAGATTCTTCTTTCACCGGCACTCAGCTATACCACAAAACATTTGCATAATGATGTGGAGAAAGA
GAGAAAGGAAAAATTGCCAAAAGAAATAGAGGAAGATAAACTAAAACGAGAAGAAGAAAAAAGAGAAGCTGAA
AAGAGTGAAGATTCCTCTGGTGCTGCAGGCCTCTCAGGCTTACATCGCACATACAGCCAGGACTGTAGCTTTAAAA
ACAGCATGTACCATGTTGGAGATTACGTCTATGTGGAACCTGCAGAGGCCAACCTACAACCACATATCGTCTGTAT
TGAAAGACTGTGGGAGGATTCAGCTGAAAAAGAAGTTTTTAAGAGTGACTATTACAACAAAGTTCCAGTTAGTAA
AATTCTAGGCAAGTGTGTGGTCATGTTTGTCAAGGAATACTTTAAGTTATGCCCAGAAAACTTCCGAGATGAGGAT
GTTTTTGTCTGTGAATCACGGTATTCTGCCAAAACCAAATCTTTTAAGAAAATTAAACTGTGGACCATGCCCATCA

GCTCAGTCAGGTTTGTCCCTCGGGATGTGCCTCTGCCTGTGGTTCGCGTGGCCTCTGTATTTGCAAATGCAGATAAA
GGTGATGATGAGAAGAATACAGACAACTCAGAGGACAGTCGAGCTGAAGACAATTTTAACTTGGAAAAGGAAAA
AGAAGATGTCCCTGTGGAAATGTCCAATGGTGAACCAGGTTGCCACTACTTTGAGCAGCTCCATTACAATGACATG
TGGCTGAAGGTTGGCGACTGTGTCTTCATCAAGTCCCATGGCCTGGTGCGTCCTCGTGTGGGCAGAATTGAAAAAG
TATGGGTTCGAGATGGAGCTGCATATTTTTATGGCCCCATCTTCATTCACCCTGAAGAAACAGAGCATGAGCCCAC
AAAAATGTTCTACAAAAAAGAAGTATTTCTGAGTAATCTGGAAGAAACCTGCCCCATGACATGTATTCTCGGAAA
GTGTGCTGTGTTGTCATTCAAGGACTTCCTCTCCTGCAGGCCAACTGAAATACCAGAAAATGACATTCTGCTTTGT
GAGAGCCGCTACAATGAGAGCGACAAGCAGATGAAGAAATTCAAAGGATTGAAGAGGTTTTCACTCTCTGCTAAA
GTGGTAGATGATGAAATTTACTACTTCAGAAAACCAATTGTTCCTCAGAAGGAGCCATCACCTTTGCTGGAAAAGA
AGATCCAGTTGCTAGAAGCTAAATTTGCCGAGTTAGAAGGTGGAGATGATGATATTGAAGAGATGGGAGAAGAA
GATAGTGAGTCTACCCCAAAGTCTGCCAAAGGCAGTGCAAAGAAGGAAGGCTCCAAACGGAAAATCAACATGAG
TGGCTACATCCTGTTCAGCAGTGAGATGAGGGCTGTGATTAAGGCCCAACACCCAGACTACTCTTTCGGGGAGCTC
AGCCGCCTGGTGGGGACAGAATGGAGAAATCTTGAGACAGCCAAGAAAGCAGAATATGAAGGCATGATGGGTGG
CTATCCGCCAGGCCTTCCACCTTTGCAGGGCCCAGTTGATGGCCTTGTTAGCATGGGCAGCATGCAGCCACTTCAC
CCTGGGGGGCCTCCACCCCACCATCTTCCGCCAGGTGTGCCTGGCCTCCCGGGCATCCCACCACCGGGTGTGATGA
ACCAAGGAGTGGCCCCTATGGTAGGGACTCCAGCACCGGGTGGAAGTCCATATGGACAACAGGTGGGAGTTTTGG
GGCCTCCAGGGCAGCAGGCACCACCTCCATATCCCGGCCCACATCCAGCTGGACCCCCTGTCATACAGCAGCCAA
CAACACCCATGTTTGTAGCTCCCCCACCAAAGACCCAGCGGCTTCTTCACTCAGAGGCCTACCTGAAATACATTGA
AGGACTCAGTGCGGAGTCCAACAGCATTAGCAAGTGGGATCAGACACTGGCAGCTCGAAGACGCGACGTCCATTT
GTCGAAAGAACAGGAGAGCCGCCTACCCTCTCACTGGCTGAAAAGCAAAGGGGCCCACACCACCATGGCAGATG
CCCTCTGGCGCCTTCGAGATTTGATGCTCCGGGACACCCTCAACATTCGCCAAGCATACAACCTAGAAAATGTTTA
G

**PBRM1 knockdown plasmid sequences**

| Clone Name | Symbol | Location | Length | Target Sequence |
|---|---|---|---|---|
| HSH105887-LVRU6GP-a(OS520058) | PBRM1 | 263 | 21 | GCCATGAACTCTATAATACCA |
| HSH105887-LVRU6GP-b(OS520059) | PBRM1 | 689 | 21 | CCATAGTTGTAGCTACAAATC |
| HSH105887-LVRU6GP-c(OS520060) | PBRM1 | 1132 | 21 | GGTCGTTTATCAGCAATTACA |
| HSH105887-LVRU6GP-d(OS520061) | PBRM1 | 3147 | 21 | GGTCATGTTTGTCAAGGAATA |

## Reference


1. Peng H, Zhang Y, Zhou Z, Guo Y, Huang X, Westover KD, et al. Intergrated analysis of ELMO1, serves as a link between tumour mutation burden and epithelial-mesenchymal transition in hepatocellular carcinoma. *EBioMedicine.* 2019;46:105-18.
2. Guo Y, Wang J, Zhang L, Shen S, Guo R, Yang Y, et al. Theranostical nanosystem-mediated identification of an oncogene and highly effective therapy in hepatocellular carcinoma. *Hepatology.* 2016;63(4):1240-55.
3. Liu J, Shen JX, He, and Zhang GJ. Bioluminescence Imaging for Monitoring miR-200c Expression in Breast Cancer Cells and its Effects on Epithelial-Mesenchymal Transition Progress in Living Animals. *Mol Imaging Biol.* 2018;20(5):761-70.
4. Xie W, Lu Q, Wang K, Lu J, Gu X, Zhu D, et al. miR-34b-5p inhibition attenuates lung inflammation and apoptosis in an LPS-induced acute lung injury mouse model by targeting progranulin. *J Cell Physiol.* 2018;233(9):6615-31.
5. Luo Y, Niu G, Yi H, Li Q, Wu Z, Wang J, et al. Nanomedicine promotes ferroptosis to inhibit tumour proliferation in vivo. *Redox Biol.* 2021;42:101908.
6. Ye X, Guo Y, Zhang Q, Chen W, Hua X, Liu W, et al. betaKlotho suppresses tumor growth in hepatocellular carcinoma by regulating Akt/GSK-3beta/cyclin D1 signaling pathway. *PLoS One.* 2013;8(1):e55615.
7. Zhang Y, He L-J, Huang L-L, Yao S, Lin N, Li P, et al. Oncogenic PAX6 elicits CDK4/6 inhibitor resistance by epigenetically inactivating the LATS2-Hippo signaling pathway. *CLINICAL AND TRANSLATIONAL MEDICINE.* 2021;11(8).
